# Systematic Review of the Effectiveness of Advocacy Interventions for Adult Victims of Domestic Violence Within an Emergency Department Setting

**DOI:** 10.7759/cureus.25599

**Published:** 2022-06-02

**Authors:** Mohamed Bushry Basheer, Rachel Bell, Adrian A Boyle

**Affiliations:** 1 Clinical School of Medicine, Cambridge University Hospitals Foundation Trust, Cambridge, GBR; 2 Emergency Medicine, Addenbrooke's Hospital, Cambridge University Hospitals Foundation Trust, Cambridge, GBR

**Keywords:** emergency department, independent domestic violence advisors, advocacy, systematic review, domestic violence

## Abstract

Advocacy interventions for survivors of domestic violence are well established and supported by evidence in some community and healthcare settings. Survivors of domestic violence identified in emergency departments have important differences, and it is not clear whether evidence can be applied to this population. We conducted an inclusive systematic review of controlled studies evaluating the effectiveness of advocacy workers for adult survivors identified in emergency departments. We identified five studies, all with substantial methodological flaws. The outcome measures were very varied. No study reported harm from advocacy. Most reported benefits from referrals to advocacy workers. Despite weak evidence, referral to advocacy workers for survivors of domestic violence is not harmful and offers benefits.

## Introduction and background

Introduction

Gender-based violence is a major issue globally, with the WHO estimating that one in three women around the world have endured physical and/or sexual intimate partner violence (IPV) or non-partner sexual violence [1]. It is a major violation of women's human rights, and doctors are rightly concerned about how best to help survivors break free of the cycle of violence. Survivors of domestic violence are present in various settings, such as the police, community-based centres, and shelters, as well as emergency departments (EDs). However, many victims are missed: the British Crime Survey found that only 12% of survivors would go to the police and only 10% would feel comfortable reporting to medical staff [2]. These data might be affected by reporting bias, especially because the survey was a self-completed questionnaire, but even so, the data are concerning.

Looking specifically at patients presenting at the emergency department (ED), studies have shown the incidence of domestic violence among women to range from 1% to 2% [3,4]. Women are also at greater risk of domestic violence than men, suffering from a greater frequency and severity of violence [2]. Survivors are also likely to present themselves to the emergency department during hours when social services relevant to them are not available [4]. There is also a mismatch between survivors presenting to the hospital and those that are brought to the police's attention; this mismatch also results in very few abusers getting convicted. A record linkage study by Boyle et al. (2005) found that 10% of domestic assaults requiring emergency department treatment were recorded by the police [3]. Survivors presenting in the hospital are more likely to have problems with drug and alcohol abuse, suicidal ideation, and other psychiatric issues [5] than community survivors.

Given the risks facing such survivors and the chances of them being missed by the police, domestic violence advocates [6] placed within the ED have been seen as a way to help survivors get justice and reduce the chance of re-admission and/or further violence and death. Evidence of benefit for advocacy workers in other community and healthcare settings is well established and is translated into guideline recommendations by the National Institute for Health and Care Excellence (NICE) [7]. The differing characteristics of survivors in emergency departments compared to survivors in the community and other healthcare settings limits the confidence that this is an effective intervention.

We aimed to evaluate the evidence for before/after studies and randomised control trials looking at the effectiveness of such advocacy work for victims presenting in the ED to determine if they reduce abuse-related co-morbidities, re-admission rates, use of community/police resources, and among other outcomes. This evidence would provide a useful background for future controlled studies.

Methods

We conducted an inclusive, systematic review of published literature. We included studies that reported primary research studies evaluating the effectiveness of advocacy workers in supporting survivors of domestic abuse conducted in emergency departments. We excluded studies that reported screening trials, interventions to increase identification of abuse by healthcare staff, studies conducted on children, and studies that weren’t reported in English.

The search for studies was done via the following online databases from their start dates till February 2022: PubMed/MEDLINE, Cochrane Library, CINAHL/Ebsco, EMBASE, and PsycINFO.

The following keywords were searched for: (“domestic violence” OR “intimate partner violence” OR “partner abuse” OR “spouse abuse” OR “abusive partners” OR “battered women” OR “abused” OR “male domestic violence*”) AND (“A&E” OR “emergency department” OR “hospital” OR “accident and emergency”) AND (“advocacy” OR “intervention” OR “IDVA” OR “independent domestic violence advis*”).

We also contacted researchers to look for unpublished studies and examined a trial registry.

For all English-language studies found via the search strategy, the abstract and methodology were reviewed for whether the study meets the selection criteria, and the bibliography of selected studies will also be looked for further studies that could have been missed. Discussions and consensus amongst the co-authors addressed uncertainties around particular studies regarding inclusion.

The Jadad scale was used to assess the quality of studies found. We anticipated that there would be few high-quality double-blinded randomised controlled trials (RCTs) and aimed to include before and after studies.

Results

We only found one randomised control trial that met our inclusion criteria and several observational studies that were judged as too weak for inclusion. We also looked for studies that compared emergency departments with access to advocacy services compared to departments that did not, but were unable to find such studies.

We found a non-peer-reviewed study done as part of a dissertation by a UK emergency consultant, as well as an unpublished randomised controlled trial carried out as part of a Ph.D. thesis.

Three hundred and fifteen records of studies were identified through database searching (Medline=190; CINAHL=38; Cochrane Library=4; PsycINFO=32; and EMBASE=51). Of these, 291 were excluded for not being relevant to this review. The remaining 24 studies' full text was assessed for eligibility, with 19 being excluded for not meeting the selection criteria. 

**Figure 1:**
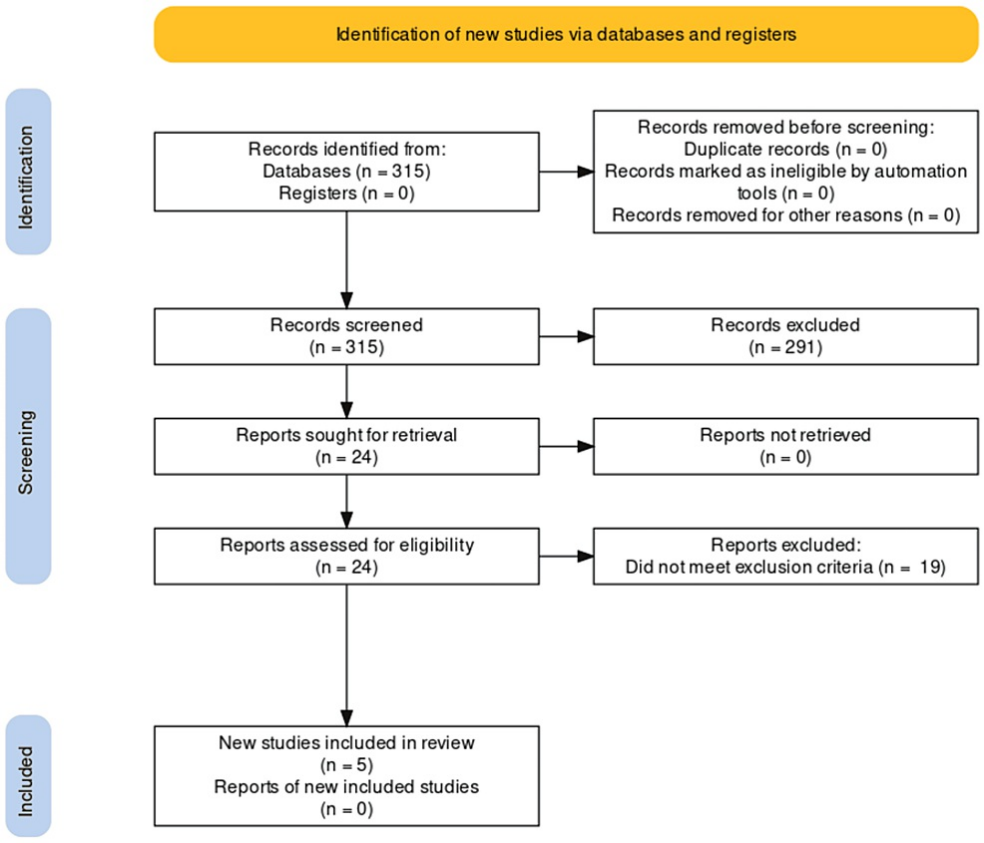
PRISMA flowchart.

In Figure 1, we have shown the PRISMA flowchart for our study selection process.

## Review

Summary of included studies

Table 1 shows a summary of the included studies.

**Table 1:** Summary of included studies. RCT: randomised control trial, IPV: intimate partner violence, IDVA: independent domestic violence advise, ED: emergency department, SSSI: standard social service intervention, PTSD: post-traumatic stress disorder.

Study	Country	Type	Outcome	Key results	Jadad score
Hyman (2002) [8]	USA	RCT has been done as part of a Ph.D. thesis, but published as a dissertation abstract	Reporting of abuse and distress. Community resource use engaging in safety behaviours	Advocacy intervention provided a little benefit over SSSI; slight decrease in “PTSD-symptomatology” for advocacy group over SSSI	2
Kendall et al. (2009) [9]	USA	Before/after study	Perception of safety. Completion of a safety plan	Improvements in outcomes, but many unable to reach for follow-up	1
Muelleman and Feighny (1999) [10]	USA	Before/after study	Use of shelters (community resource use). Repeat police calls full orders of protection. Repeat ED visits for domestic violence	Access to meeting an advocate increased shelter use/community resource use but had no statistically significant effect on repeat police calls, full orders of protection, or repeat ED visits for domestic violence	0
Halliwell et al. (2019) [11]	UK	Before/after study; used community-based IDVAs as comparison	Health outcomes for survivors; risk of being harmed cost-savings	Chance of safety (calculated using severity of abuse grid filled by IDVAs) for survivors increased twofold if hospital survivors received continued contact with IDVA in ED. Reduction in cost of IPV survivors presenting to ED offsets cost of producing IDVAs	0
Williamson and Boyle (2012) [12]	UK	Before/after study; a service evaluation is done as part of a dissertation	Number of repeat ED attendances. Risk of death and other injuries	Access to IDVAs resulted in improvements across all outcomes	0

Discussion

We found a few studies that were suitable for inclusion in this review. Many studies have been done looking at the effectiveness of advocacy work in the community, but very little looking at the emergency department. Four before/after studies were selected as well as an unpublished randomised control trial is done as part of a Ph.D. thesis. We contacted leading authorities on domestic violence to try to get the full text of the thesis discussing the randomised control trial, but were unable to access it and had to rely on the published abstract and an online preview.

There was a wide variation in the reported outcomes, such as abuse-related morbidities, including fractures, other injuries, and death, as well as subsequent emergency department admissions due to further abuse, patient use of the resources available such as shelters and community resources, and police and judicial action, among others. The heterogeneity of outcomes does make comparisons difficult, further diluting the quality of evidence for advocacy work, but broadly, all studies included looked at indicators of victim safety, as well as having broad agreement on what an “advocacy worker” does: coming up with a safety plan with the survivor and acting as a bridge to the wider community and judicial services.

Halliwell et al. (2019) carried out a quantitative evaluation of IDVA services based in both the emergency department and in the community in the UK, with comparisons being made between the two [11]. Five hospital IDVA services were looked at, with the outcomes including physical and mental health outcomes as well as the use of community and health services. Hospital survivors of domestic violence were also more likely to have complex needs and vulnerabilities, such as suffering from drug and alcohol abuse, financial difficulties, as well as being more likely to be disabled. Survivors presenting in the hospital were also more likely to have suicidal ideation or have attempted suicide in the past. While community IDVA services were more likely to report cases as being at high risk for harm, the patients seen by hospital IDVAs were likely to be victims of severe sexual abuse. While patients seen by community IDVAs were more likely to have been in touch with the police, hospital patients were, unsurprisingly, more likely to have come in for injuries or mental ill-health specifically linked to experiencing domestic violence. While there were small differences in the types of referrals and help provided by community vs hospital-based IDVAs, largely there was similarity in providing support with safety planning, health and wellbeing, and accessing police services and housing. In terms of outcomes, hospital IDVAs were more likely to work with patients who had poorer physical and mental health compared to the general population; these health outcomes did not largely improve after having access to IDVAs, but there were declines in PTSD symptomology and reports of mental health concerns such as anxiety and depression. In terms of domestic violence-related outcomes, however, the evaluation found more positive news in the sense that hospital survivors accessing IDVAs were more likely to experience a cessation of abuse compared to those accessing community-based IDVAs, including reductions in physical abuse, sexual abuse, harassment and stalking, and jealous, coercive, and controlling behaviours. The longer that survivors presenting in the emergency department had access to IDVAs, the greater the chance of using wider community services and subsequent feelings of safety and cessation of abuse. Due to the subsequent reductions in the use of health services, Halliwell et al. estimated an overall cost-savings of £2050 per patient per year for the hospital-based advocacy programme. Another benefit of the ED-based advocacy programme identified by Halliwell et al. was that “hidden” survivors were more likely to be identified by hospital IDVAs, e.g., those who were older, from higher-income households, and survivors who were pregnant. The cost savings as well as accessing parts of the population that would otherwise suffer in silence means that Halliwell et al. conclude that ED-based advocacy programmes could be an efficient use of health care funding. However, the authors do acknowledge the limitations of their non-experimental technique, with a Jadad score of 0 being assigned to their study design. This was not a randomised control trial, and many of the domestic violence outcomes measured were self-reported and likely subject to reporting bias.

Muelleman and Feighny (1999) was a much older study, conducted in the emergency department of a hospital in Missouri, USA [10]. This was a before and after study, having many of the same methodological flaws as four out of the five studies included in this review. A sample of 105 women (>18 years of age) who presented to the ED having been injured due to domestic violence were asked to meet with an advocacy worker. These women's outcomes were measured by looking at their use of shelters, use of counselling available at women's shelters, as well as the number of repeat police calls, subsequent ED attendances, as well as how many managed to get court orders of protection. After a study participant arrived in the ED and was consented to, an advocate would arrive to discuss what happened with the patient, safety planning, as well as educate patients on the cycle of violence and the resources available within the community, such as shelters and counselling. Muelleman and Feighny found that the use of shelters and shelter-based counselling increased significantly from 11% to 28% and 1% to 15% of the cohort, respectively, following the meeting with the advocacy worker, suggesting that some victims were not effectively using community resources. But by bridging access to community resources with the ED via an advocacy worker, the use of such resources could be increased. However, the authors found no statistically significant change in repeat police, getting full orders of protection, nor on repeat ED visits for domestic violence; the study was done over a six-month period, with women recruited if they had experienced domestic violence in a six-month before period. In addition, despite increases in community resource use, the proportion that used them was only a minority. While Muelleman and Feighny report advocacy interventions to be of some benefit, the weak nature of evidence from a before/after design means that, overall, the evidence from this study for advocacy programmes being an effective intervention is weaker compared to Halliwell et al. (2019).

Kendall et al. (2009) was also a before-and-after study that had patients, both male and female, identified as survivors of domestic violence meet with an ED-based advocacy worker who would come up with a safety plan as well as point to wider community resources available [9]. This study was done over almost two years, from 2002 to 2004. After ED intervention, follow-up was done at two, six, and 12 weeks to determine progress in implementing the safety plan as well as to ask the patients which community resource referrals they found most helpful. However, the authors experienced many losses to follow-up, with less than 1% of patients responding at the 12-week follow-up, and so decided to call at two days post-intervention as well. About 97% of the survivors identified were female, and 3% were male. However, the authors state that patient screening and inclusion into the study were flawed, with up to 30% of patients during random chart reviews found to not have been screened during triage; the number of patients who were screened positively but refused ED intervention was also not known. Of those who followed up, the overwhelming majority, 96-98% depending on the number of weeks post-intervention, stated they were safer as a result of the ED-based advocacy intervention. A mean percentage of 49-59% of patients also completed their safety plans across the various follow-up stages. The most beneficial resource referral mentioned by the patients during follow-up was the police, followed by counselling, legal help, and domestic abuse shelters. While the authors concluded that their study showed a positive impact on domestic violence survivors from the advocacy intervention, the quality of the evidence is very weak, as well as is being compounded by the self-reported outcomes included in this study. The Jadad score of 1 for this study was due to its thorough description of withdrawals and drop-outs, but this study's data is overall not reliable for making conclusions about the effectiveness of advocacy interventions. Those who were lost to follow-up may also have been those who felt the programme did not help them, and so further bias would have been introduced into the study.

Williamson and Boyle (2012) were the final before and after studies included in this review [12]. This study was done in the UK as part of a dissertation, and the full text was obtained after contacting the authors. Forty-five female patients were included in the study, and while the IDVA was based at the local county council, 38 of the patients were referred from the emergency department. Of the 45, only 40 were eligible for the IDVA programme since five were not in the catchment area, but only 25 could be contacted, of which 84% (21) engaged with the service. The outcomes looked at in this before and after study were total ED attendances and attendances per patient-year, as well as health outcomes such as musculoskeletal injury, head injury, stabbing, overdose, and, from these figures, the associated risk of death. The concerning finding was that the majority of patients identified as domestic violence survivors in the ED were at high to very high risk of serious injury or death (coordinated action against domestic abuse (CAADA)/domestic abuse, stalking and harassment (DASH) risk assessment tool). However, the high engagement indicates the acceptability of the IDVA programme. Fewer than half of identified survivors were also known to the police, underlying the importance of targeting survivors presenting in ED as found by Boyle et al. [3]. The study found improvements across all outcomes; a reduction in ED attendance as well as injuries. However, the short time period the study looked at, ranging from a few days to 10 months depending on the patient, as well as the before and after design and small sample size, makes the quality of evidence quite weak and subject to findings being influenced by regression to the mean.

From the four before and after studies considered above, which collectively provide weak evidence compared to randomised control trials, the conclusion would appear to be that advocacy interventions in ED are helpful in bridging “hidden” victims who might have been missed by community services and law enforcement with important resources such as shelters and counselling. It also appears to be that IDVAs are helpful in increasing patient safety and reducing costs to the health service due to reductions in subsequent hospitalisation. However, before and after studies are non-randomised and provide weak evidence due to the risk of confounding as well as biases [13], and discussed in this review. Therefore, the best form of evidence would be a cluster randomised control trial, comparing EDs with and without IDVAs to determine the effectiveness of advocacy. In our literature search, we only came across one randomised control trial that met our inclusion criteria; this was a trial done as part of a Ph.D. by Hyman (2002).

Since we could not access the full text of Hyman (2002), we had to rely on the abstract and a preview available via ProQuest [8]. This was a trial looking at adult female survivors of domestic violence who were screened in an ED and then randomised to either an advocacy worker or the standard social service intervention. Since we could not access the full text, we could not find what was meant by “standard social service intervention”. The outcomes looked at in the trial were the use of community resources, engagement in safety behaviours, cessation of abuse, and progression through the stages of change outlined within the transtheoretical model of behaviour change [14]. The trial found improvements in outcomes across both groups, with no significant difference between the two treatments. There was a greater decrease in PTSD symptomatology in the group accessing IDVA services, but this was not statistically significant (p=0.06). The study did find specific clusters among the population looked at, which could be differentiated using factors such as the abuser's relationship to children and level of distress; movement through the stages of change could be influenced by these clusters, and therefore, looking at these subgroups for specific interventions could be a promising role for future research. However, for the purposes of this systematic review, advocacy work itself did not appear to provide a meaningful benefit.

None of the studies included specifically evaluated male domestic violence survivors; while male survivors are in the minority, it is likely that the true number of cases is under-reported, and further research needs to be done into what sort of interventions can best help male survivors.

Before and after studies will often show a benefit, perhaps due to factors such as regression to the mean, which then disappear when a randomised control trial that reduces such biases is done. However, this systematic review cannot entirely dismiss advocacy work due to the lack of sufficient trials. Halliwell et al. (2019) also suggest potential cost-savings to hospitals as a result of advocacy work, and while this was also a before and after study, there does not seem to be any harm in advocacy work, while there may be potential benefits. A wider systematic review looking at community-based advocacy interventions by Rivas et al. (2015) also found some benefits, although the quality of evidence was also weak and meta-analysis was hindered due to the heterogeneity of studies looked at [15]. There are also potential knock-on benefits of advocacy workers that have not been looked at in our systematic review. For example, Dheensa et al. (2020) conducted a qualitative evaluation (which did not meet our inclusion criteria) of hospital-based advocacy, and from their interviews, they found that other healthcare professionals found IDVAs invaluable, one reason being that it improved their own confidence and knowledge when it came to dealing with survivors of domestic violence [16]. Furthermore, referral to advocacy is only a small part of a number of interventions that are effective for victims of domestic violence, such as staff training, creating a disclosing environment, and pathway development. The availability of an advocacy worker is likely to improve the effectiveness of all of these.

## Conclusions

Advocacy interventions for adult victims of domestic violence are not harmful. The cost-effectiveness is uncertain but likely to be beneficial. We can make a weak recommendation that advocacy interventions are likely to be helpful, unlikely to be harmful, but are of uncertain cost-effectiveness. Future research should define the meaning of outcomes, and controlled trials evaluating referral to advocacy are justified. In conclusion, survivors of domestic violence who present to emergency departments are likely to benefit from referrals to advocacy workers.
